# Reversible Central Adrenal Insufficiency Without Identifiable Cause: A Diagnostic Challenge

**DOI:** 10.7759/cureus.97505

**Published:** 2025-11-22

**Authors:** Rim Masri, Racha Farhat, Hiba Jida, Ghassan Kdouh

**Affiliations:** 1 Endocrinology, Diabetes and Metabolism, Lebanese University Faculty of Medicine, Hadat, LBN

**Keywords:** adrenal hormone recovery, hydrocortison replacement therapy, hypothalamic-pituitary-adrenal axis, reversible central adrenal insufficiency, triggers

## Abstract

Reversible central adrenal insufficiency (CAI) is a rare disorder characterized by transient impairment of cortisol secretion due to reduced adrenocorticotropic hormone (ACTH) production. It is commonly associated with exogenous glucocorticoid use, acute illness, or certain medications; however, transient CAI can also occur without identifiable triggers. We report the case of a 45-year-old female patient with transient CAI in whom no systemic illness, medication exposure, or pituitary abnormality was identified. Laboratory evaluation revealed a markedly low morning cortisol level (1.3 µg/dL) and suppressed plasma ACTH (3.13 pg/mL). Pituitary magnetic resonance imaging (MRI) was unremarkable, and other pituitary hormones were within reference ranges. The patient received hydrocortisone therapy led to rapid symptomatic improvement and normalization of cortisol levels within three months, after which treatment was discontinued. Long-term follow-up showed sustained recovery of adrenal function. This case highlights the potential for transient CAI without apparent precipitating factors and emphasizes the importance of careful diagnostic evaluation and follow-up. Early recognition, appropriate treatment, and periodic reassessment are essential to guide therapy and confirm recovery. Recognizing such idiopathic presentations can help prevent misdiagnosis and unnecessary lifelong glucocorticoid therapy.

## Introduction

Reversible central adrenal insufficiency (CAI) is a rare endocrine disorder characterized by temporary cortisol deficiency due to reduced adrenocorticotropic hormone (ACTH) or corticotropin-releasing hormone (CRH) secretion from the pituitary or hypothalamus, respectively [1,2]. The prevalence of CAI is estimated at 150-280 cases per million, though reversible or idiopathic forms are particularly uncommon and often underrecognized [1-3]. Once the inciting cause is resolved, adrenal function typically returns to normal [2,3]. Common precipitating factors include prolonged exogenous glucocorticoid exposure, which suppresses the hypothalamic-pituitary-adrenal (HPA) axis [1], and critical illnesses such as severe sepsis [4]. However, adrenal insufficiency in sepsis is not caused only by pituitary dysfunction but also by inflammatory mediators and other factors, hence the term relative adrenal insufficiency [4]. Certain medications, including immune checkpoint inhibitors, fluconazole, and retinoids, are also known to transiently suppress the HPA axis, leading to reversible CAI [5-7]. Recently, transient or reversible CAI has been reported following COVID-19 infection, likely related to hypothalamic or pituitary inflammation [8].

Distinguishing central from primary adrenal insufficiency is clinically important, as the former may resolve once the underlying cause is corrected [2,3]. This distinction is particularly significant because transient forms of CAI can mimic permanent adrenal failure, leading to unnecessary long-term glucocorticoid therapy if not accurately identified [3,9]. Diagnosis requires demonstrating low cortisol with inappropriately low or normal ACTH, often confirmed by dynamic testing such as the ACTH stimulation or insulin tolerance test [1]. Early recognition and intervention are crucial to prevent long-term complications and restore adrenal function.

In this report, we present a case of transient CAI that occurred without any identifiable cause, with recovery observed after three months of glucocorticoid therapy. This case underscores the diagnostic challenges and clinical importance of recognizing reversible forms of HPA axis dysfunction.

## Case presentation

A 45-year-old previously healthy woman was referred for endocrine evaluation after incidental thyroid nodules were discovered on ultrasound. She was a nonsmoker, with no history of alcohol or drug use, and no relevant family, genetic, or psychosocial history. The patient reported no history of pregnancy-related complications such as postpartum hemorrhage, severe hypotension, or lactation failure. Ultrasound revealed a moderately enlarged gland with bilateral nodules, including three hypoechoic nodules (4, 5, and 6 mm) in the right lobe (47 × 17 × 18 mm) and a 16 mm mixed echogenicity nodule in the left lobe (46 × 17 × 17 mm). The carotid and jugular veins were normal, and no cervical lymphadenopathy was noted. Thyroid function tests were not available at that time. The patient was started on levothyroxine 25 mcg daily by her primary care physician, despite the absence of documented biochemical hypothyroidism.

Three months later, she developed postprandial hypoglycemia, fatigue, palpitations, and dyspnea. She denied dysphagia or other compressive symptoms and reported no recent illness, medication use (especially steroids or opioids), or prior surgery. Her menstrual cycles, previously regular, became oligomenorrhea in the preceding three months.

**Figure 1:**
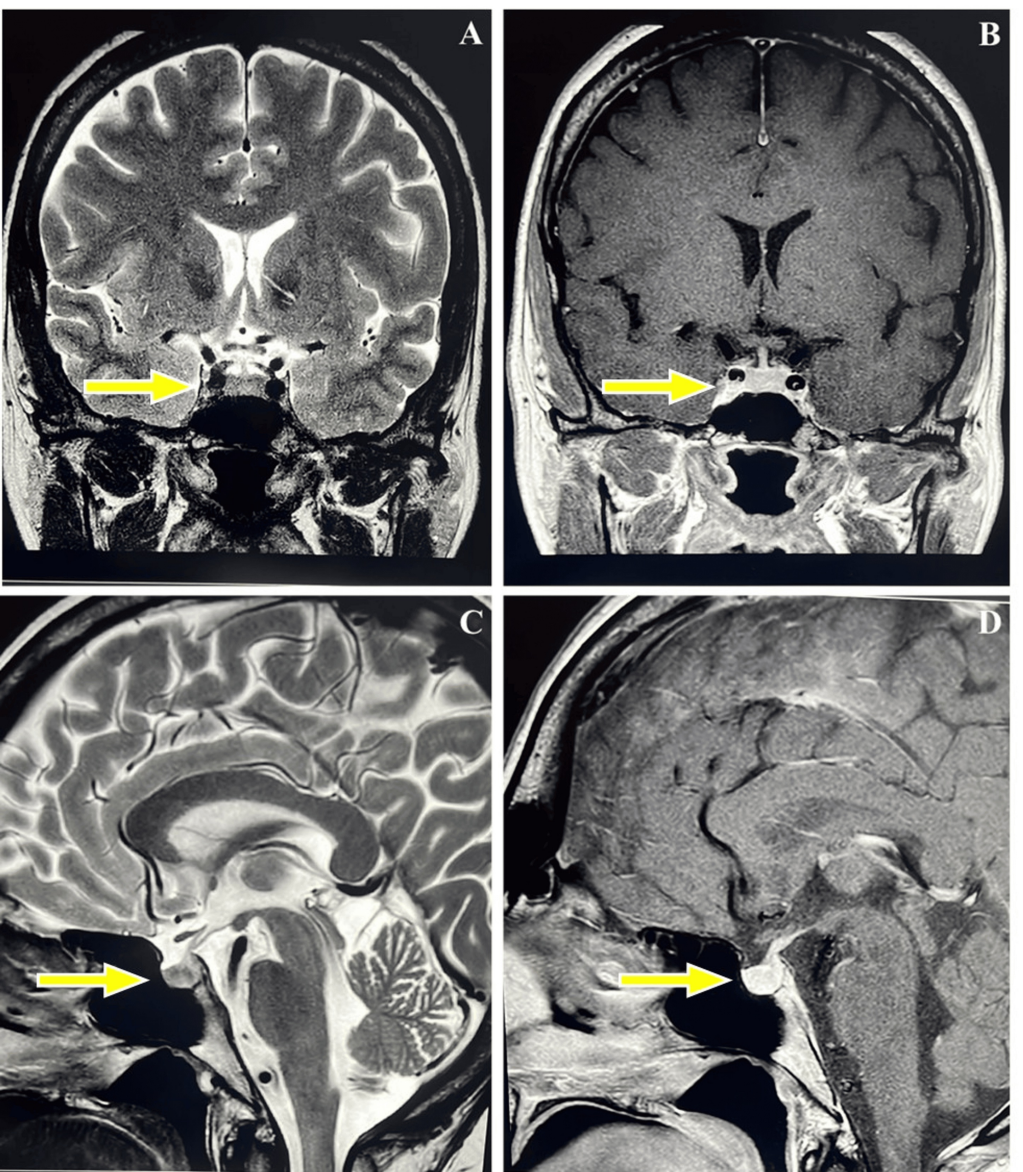
Normal MRI brain findings (TSE protocol, 2 mm slice thickness), confirming the absence of pituitary abnormalities such as mass lesions, stalk thickening, or gland enlargement TSE: turbo spin echo (A) T2 TSE coronal plane; (B) T1 TSE with contrast, coronal plane; (C) T2 TSE sagittal plane; (D) T2 TSE with contrast, sagittal plane The yellow arrow indicates the pituitary gland

Laboratory evaluation revealed morning serum cortisol of 1.3 mcg/dL and plasma ACTH of 3.13 pg/mL, consistent with CAI. Eosinophilia (>3%) and borderline hyponatremia (135 mmol/L) were also noted. Additional tests demonstrated a fasting plasma glucose of 88 mg/dL, hemoglobin A1c of 4.8%, low-density lipoprotein cholesterol of 136 mg/dL, triglycerides of 142 mg/dL, calcium of 8.8 mg/dL, serum 25-hydroxyvitamin D (25-OHVitD) of 8 ng/mL, and ferritin of 7 ng/mL. Thyroid function tests showed a thyroid-stimulating hormone (TSH) of 1.57 mcIU/mL and free thyroxine (FT4) of 1.28 ng/dL with negative antibodies. Serum prolactin was mildly elevated at 62.7 ng/mL, and macroprolactin assessment was indeterminate, though the biologically active (monomeric) prolactin was normal (Table 1). Pituitary magnetic resonance imaging (MRI) was normal, showing no mass lesions, stalk thickening, or pituitary enlargement (Figure 1). Cardiology evaluation, including a treadmill stress test, was unremarkable.

**Table 1:** Laboratory test results at initial presentation and at three-year follow-up WBC: white blood cell count; RBC: red blood cell count; MCV: mean corpuscular volume; 25-OHVITD: 25-hydroxy vitamin D; FBS: fasting blood sugar; HbA1c: hemoglobin A1c; TC: total cholesterol; HDL: high-density lipoprotein; LDL: low-density lipoprotein; TG: triglycerides; SGPT: serum glutamate pyruvate transaminase; CPK: creatine phosphokinase; ACTH: adrenocorticotropic hormone; TSH: thyroid-stimulating hormone; FT4: free thyroxine; anti-TPO: anti-thyroid peroxidase antibody; anti-TG: anti-thyroglobulin;  IGF-1: insulin-like growth factor 1; PRL: prolactin; FSH: follicle-stimulating hormone; IGF-1: insulin-like growth factor 1 This table summarizes key hematological, biochemical, and endocrine parameters measured at the time of initial presentation and again three years later. Reference (normal) ranges are provided for context. Values in bold indicate results outside the normal range, which may reflect underlying physiological or pathological changes. A dash (–) signifies that the result was not available at the time of testing

Test	Initial presentation	At three years	Normal range	Units
WBC	9.84	9.7	3.70-10.1	10^3^/uL
Neutrophils	52.1	53.5	39.3-73.7	%
Lymphocytes	34.1	35.3	18.0-48.3	%
Monocytes	8.68	7.8	4.40-12.7	%
Eosinophils	3.97	2.9	0.600-4.30	%
Basophils	1.15	0.5	0.00-2.00	%
RBC	4.88	5.15	4.00-5.50	10^6^/uL
Hemoglobin	12.7	11.3	11.0-15.0	g/dL
Hematocrit	39.2	35.5	35.0-46.0	%
MCV	80.3	68.9	80.0-96.0	fL
Platelet	356	388	140.0-424.0	10^3^/uL
Ferritin	7	5.7	10-20	ng/mL
Sodium	135	-	135-148	mmol/L
Potassium	4.0	-	3.5-5.5	mmol/L
Chloride	103	-	95-112	mmol/L
Magnesium	-	2.04	1.6-2.5	mg/dL
Calcium	-	9.7	8.4-10.5	mg/dL
Phosphorus	-	4.9	2.5-5	mg/dL
Total protein	-	7.4	6.4-8.3	g/dL
Albumin	-	4.3	3.5-5	g/dL
25-OH Vit D	8	28	30-60	ng/mL
Vitamin B12	-	508	187-1100	pg/mL
FBS	88	103	70-100	mg/dL
HbA1c	4.8	5.7	4.5-5.7	%
Insulin level	17.8	-	4.8-27.7	mcU/mL
Uric acid	3.9	-	2.5-6.5	mg/dL
TC	216	125	<200	mg/dL
HDL	66	38	>35	mg/dL
LDL	136	71	<130	mg/dL
TG	142	130	40-160	mg/dL
SGPT	19	13	0-55	U/L
CPK	34	69	29-168	IU/L
Cortisol	1.3	10.7	3.7-19.4	mcg/dL
ACTH	3.13	-	7-63	pg/mL
TSH	1.57	1.70	0.35-4.94	mcIU/mL
FT4	1.28	-	0.93-1.71	ng/dL
Anti-TPO	14.5	-	Negative < 34	UI/mL
Anti-TG	19.4	-	Negative < 115	IU/mL
PRL	67.1	41.2	5-23	ng/mL
MacroPRL				
Monomeric (postprecipitation)	16.59	-	5-23	ng/mL
Recuperation	42.62	-	<40%, presence of macroPRL; 40-65%, indeterminate; >65%, absence of macroPRL	%
FSH	3.05	36.8	Follicular, 3.03-8.08; luteal, 2-8	IU/L
IGF-1	155.2	-	-	-

Dynamic endocrine testing was not performed because basal cortisol and ACTH concentrations were unequivocally low in the context of compatible clinical symptoms, providing sufficient biochemical confirmation of CAI. Autoimmune screening (antinuclear antibody (ANA) and extractable nuclear antigen (ENA) panels) was not performed.

A diagnosis of CAI was established. Hydrocortisone replacement therapy was initiated at a dose of 10 mg in the morning (8 AM), 5 mg at noon, and 5 mg at four o'clock in the afternoon (4 PM), along with vitamin D and iron supplementation. Levothyroxine was discontinued. Six weeks later, morning serum cortisol increased to 14 mcg/dL. TSH remained stable at 1.45 mcIU/mL, and FT4 was 1.11 ng/dL. Ferritin increased to 35 ng/mL, and 25-OHVitD to 15 ng/mL. Her symptoms significantly improved, and her menstrual cycles also resumed. The hydrocortisone dose was subsequently tapered to 10 mg AM, 5 mg noon, and 2.5 mg 4 PM.

**Figure 2:**
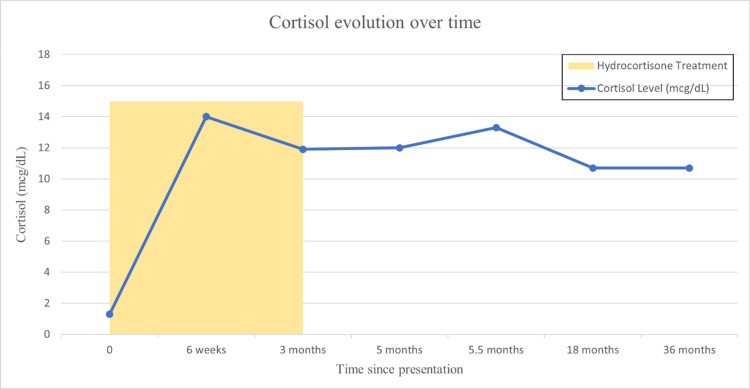
Cortisol levels over time following presentation and hydrocortisone treatment. The graph demonstrates progressive and sustained recovery of adrenal function after hydrocortisone tapering and discontinuation, confirming transient central adrenal insufficiency Cortisol levels (mcg/dL) increased markedly after initiation of hydrocortisone therapy (shaded area), peaking at six weeks, followed by normalization. After cessation of treatment, cortisol levels remained within the normal range and stabilized over the subsequent months, indicating sustained adrenal recovery up to 36 months postpresentation

At three months, repeat laboratory testing demonstrated a morning cortisol of 11.9 μg/dL and TSH of 0.9 mcIU/mL. Repeat thyroid ultrasonography showed stable nodules. Hydrocortisone therapy was progressively tapered off. Serial morning cortisol measurements over two months (obtained at four-week intervals) remained within the normal range (12.0 mcg/dL and 13.3 mcg/dL) (Figure 2). The patient remained clinically well without steroid replacement therapy. These findings were consistent with transient CAI, likely attributable to functional suppression of the HPA of uncertain etiology. Although dynamic endocrine testing (such as the ACTH stimulation test) was not performed, the pattern of serial cortisol recovery, together with progressive clinical improvement, confirmed the diagnosis of transient CAI (Figure 2).

In summary, the patient initially presented with fatigue, postprandial hypoglycemia, and dyspnea that developed progressively over three months. Following initiation of hydrocortisone replacement, symptoms improved within six weeks, with normalization of cortisol levels and menstrual function. Full adrenal recovery was achieved by three months and maintained at three-year follow-up.

**Figure 3:**
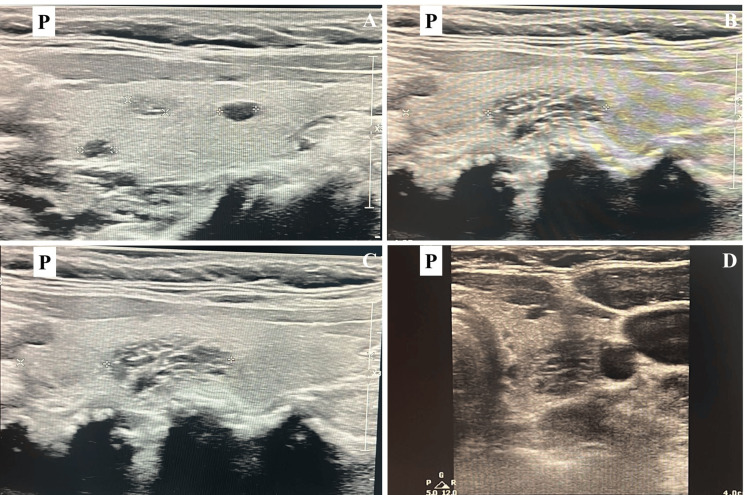
Repeated thyroid ultrasound showing stable nodules in both lobes. The consistent size and echogenicity of the nodules over three years indicate benign and nonprogressive thyroid disease (A) Right thyroid lobe: three stable hypoechoic nodules measuring 4 mm, 5 mm, and 6 mm with regular contours. (B, C, D) Left thyroid lobe: stable mixed-echogenicity oval nodule measuring 16 mm with regular margins. The “+” signs mark the boundaries of each nodule for measurement

At three years (36 months), morning cortisol was 10.7 μg/dL (Figure 2). The patient remained off both hydrocortisone and levothyroxine (Table 1). The thyroid ultrasound identified stable thyroid nodules in both lobes (Figure 3). The patient had a favorable long-term prognosis, with full recovery of adrenal function. 

## Discussion

CAI is an uncommon endocrine disorder characterized by impaired cortisol production secondary to inadequate secretion of ACTH. Well-established causes of CAI include pituitary or hypothalamic neoplasms, infiltrative diseases, traumatic brain injury, surgery, radiation, and prolonged exogenous glucocorticoid exposure [1,2]. Table 2 summarizes the major etiologies and relative prevalence of acquired CAI among high-risk populations [3].

**Table 2:** Etiology and prevalence of acquired central adrenal insufficiency (CAI) A dash (–) signifies that the result was not available at the time of testing Source: [3]

Category	Cause	Prevalence/notes
Sellar mass	Craniopharyngioma	87%
	Pituitary adenoma (secreting and non-functioning)	Up to 40% preoperatively, 75% postoperatively
	Pituitary carcinoma or metastases	-
	Other skull base tumors	-
Drug induced	Withdrawal of exogenous glucocorticoids	7% (asthma with inhaled glucocorticoids) to 60% (hematologic malignancies)
	Surgery for Cushing’s syndrome	Up to 100% (recovers within 3 to 24 months)
	Immune checkpoint inhibitors (e.g., ipilimumab)	Up to 20%
Post-intracranial procedures	Pituitary or intracranial surgery	Up to 50%
	Pituitary irradiation	12-68% (increases over time)
	Cranial/total-body irradiation for non-pituitary tumors	Up to 10%
Infiltrative	Neurosarcoidosis	Up to 49%
	Histiocytosis	Up to 10%
	Hemochromatosis	Up to 45%
Inflammatory	Hypophysitis	Up to 60%
	Meningitis (particularly tuberculosis)	-
Traumatic/vascular	Pituitary apoplexy	-
	Traumatic brain injury	Up to 8%
	Subarachnoid hemorrhage	Up to 6%
Miscellaneous	Idiopathic	-
	Empty sella syndrome	Up to 15%
	Relative adrenal insufficiency (acute illness/septic shock)	10% (hospitalized) to 60% (septic shock)
	Liver cirrhosis (all stages)	10-82% depending on test used

**Figure 4:**
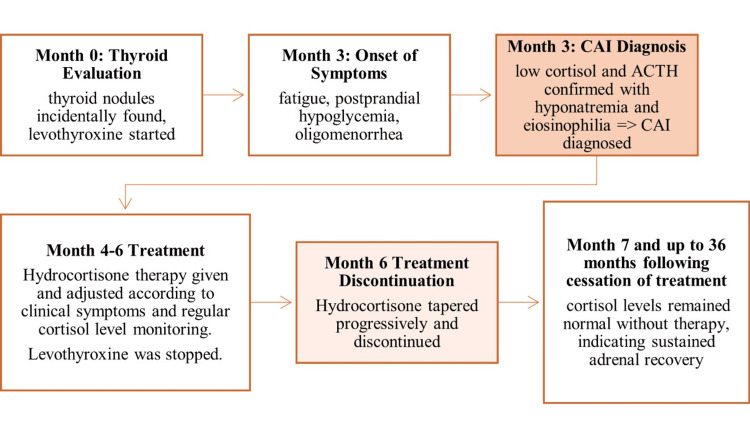
Timeline of clinical course, interventions, and outcomes in the reported case of transient central adrenal insufficiency (CAI) The figure illustrates the temporal relationship between symptom onset, diagnosis, treatment, and recovery

The present case highlights an unusual instance of transient, idiopathic CAI in an otherwise healthy woman, emphasizing the need to consider reversible or functional etiologies. The diagnosis was supported by low cortisol and ACTH levels, along with indirect biochemical findings such as mild eosinophilia and borderline hyponatremia. Dynamic testing, such as the ACTH stimulation test, was not performed because basal cortisol and ACTH levels were unequivocally low in conjunction with compatible clinical features. In this case, the progressive normalization of endogenous cortisol, which paralleled clinical recovery following glucocorticoid therapy, with complete adrenal function restoration by three months and sustained normalization at three years, thereby confirmed the diagnosis. The temporal sequence of biochemical and clinical recovery is summarized in Figure 4 (Appendices). Although the patient fully recovered with short-term glucocorticoid replacement, the exact mechanism remains uncertain; transient hypothalamic-pituitary axis dysfunction related to an immune-mediated or subclinical viral process cannot be excluded.

Although critical illness-related corticosteroid insufficiency (CIRCI) is a well-recognized and extensively described cause of transient HPA axis dysfunction, particularly in the intensive care unit (ICU) or during septic shock [4], our patient had no systemic illness or acute stressors. Nonetheless, CIRCI demonstrates that the HPA axis is vulnerable to transient suppression in acute or subacute stress states, which may occur without overt symptoms.

One of the most common causes of CAI, or secondary adrenal insufficiency, is the prolonged exogenous use of corticosteroids, particularly when doses exceed 25 mg/day of hydrocortisone or 6 mg/day of prednisolone for over three to four weeks [9]. Suppression of the HPA axis may follow systemic, topical, inhaled, intra-articular, and ocular steroid administration. In patients with acute exacerbation of chronic obstructive pulmonary disease (COPD) treated with a 14-day corticosteroid regimen (starting with 40 mg of methylprednisolone followed by 13 days of 40 mg prednisolone), HPA axis suppression occurred in up to 89% of cases, with persistent suppression in 33% of patients even three weeks after discontinuation [10]. Notably, even brief courses of supraphysiologic steroid doses can trigger transient CAI, with the potential for prolonged suppression [11,12]. These observations emphasize the risk of HPA axis dysfunction even with short-term steroid therapy.

Opioid-induced adrenal insufficiency is another known iatrogenic form of CAI. Buprenorphine, a partial mu-opioid receptor agonist, has been shown to suppress ACTH and cortisol levels via HPA axis inhibition [13,14]. Hypothyroidism is an underlying endocrine factor in cases of transient CAI. Chronic hypothyroidism may induce pituitary hyperplasia due to elevated thyrotropin-releasing hormone, impairing corticotrope function and HPA axis integrity. A case has been reported of reversible CAI in the setting of primary hypothyroidism and systemic retinoid therapy [7]. Another case described empty sella and CAI in treated hypothyroidism, further supporting this association [15]. In our case, the patient was found to have a multinodular goiter on thyroid ultrasound and transient hypothyroidism, which was managed with levothyroxine initiated by her primary care physician. Autoimmune thyroiditis was unlikely, as thyroid peroxidase and thyroglobulin antibodies were negative. Rathke’s cleft cysts, although usually asymptomatic, can present with transient CAI. Surgical intervention can reverse adrenal dysfunction, as shown in one case where transsphenoidal surgery eliminated the need for glucocorticoids [16].

In our patient, no steroid, opioid, systemic, or critical illness was identified, ruling out common reversible causes of CAI. She had a history of multinodular goiter, yet presented with symptoms (fatigue, hypoglycemia, dyspnea, palpitations) and biochemical evidence of CAI (low cortisol and ACTH). Thyroid function tests were normal, and pituitary MRI was unremarkable; other pituitary axes were intact.

Regarding possible mechanisms, mild hyperprolactinemia was noted, with normal biologically active prolactin, and the elevated total prolactin is likely due to macroprolactin, though technically in the indeterminate zone. This may reflect transient HPA dysfunction, potentially secondary to subtle hypothalamic dysregulation or stalk effect, possibly related to mild immune or inflammatory processes not evident on imaging, as has been suggested in other cases of functional pituitary impairment [2,17]. Functional suppression or idiopathic hypophysitis, even without radiological evidence, could account for this transient hormonal deficit, as functional HPA axis suppression without imaging findings has been previously described in transient hypophysitis [17]. The patient responded promptly to glucocorticoid replacement, and adrenal function recovered fully within three months, permitting successful cessation of therapy. This clinical course, which underscores the diagnostic complexity of such cases, is consistent with a transient and reversible form of CAI, a phenomenon that remains poorly understood and infrequently reported [3].

Comparable cases of transient or idiopathic CAI described in the literature share similar features, including low basal cortisol and ACTH levels, absence of structural pituitary abnormalities, and spontaneous recovery within months [5-8,10-20]. The clinical and biochemical trajectory observed in our patient aligns with these reports, supporting the concept of reversible hypothalamic-pituitary dysfunction as a distinct, albeit uncommon, entity. The data in this case are well-documented and clearly presented, strengthening the validity of the conclusions; however, the analysis remains primarily descriptive owing to the single-case design. Future studies or case series could further clarify the mechanisms involved by incorporating confirmatory dynamic testing and longitudinal comparison with similar transient CAI presentations.

Growing literature suggests that subclinical infections, mild systemic inflammatory states, or immune-mediated processes may cause reversible ACTH deficiency [3,5]. In particular, lymphocytic hypophysitis, often associated with pregnancy, anti-pituitary antibodies, and certain radiological findings (e.g., homogeneous pituitary enlargement, stalk thickening), can result in transient ACTH deficiency [17]. The reversibility of pituitary deficits, including ACTH, has been well-described in these contexts, as shown by Mori et al. [18]. Additionally, some cases may lack definitive imaging findings but still represent autoimmune hypophysitis [17].

Immune checkpoint inhibitors (e.g., combined anti-CTLA-4 and anti-PD-1 therapy) have been linked to transient CAI due to hypophysitis, with recovery following treatment cessation [5]. Chemotherapy regimens, such as cyclophosphamide, doxorubicin (also called hydroxydaunorubicin), vincristine (oncovin), and prednisone (CHOP) for diffuse large B-cell lymphoma, have also been associated with transient HPA suppression [12]. Furthermore, infectious and postviral etiologies have been implicated, with a 24-month longitudinal study showing that up to 14% of Coronavirus disease 2019 (COVID-19) survivors developed reversible CAI during the acute phase, highlighting a potential postviral etiology [8]. Subclinical viral infections or mild systemic inflammation may also transiently affect the HPA axis, although the precise mechanisms remain speculative [3].

Among medications, high-dose fluconazole has been associated with transient CAI by inhibiting adrenal steroidogenic enzymes, particularly CYP11B1 (11β-hydroxylase) and CYP17 (17α-hydroxylase), both critical for cortisol synthesis. This enzyme inhibition leads to reduced cortisol production, with adrenal function typically recovering within days to weeks after discontinuation of the drug [6]. While no definitive infectious or inflammatory trigger was identified in our patient, a subclinical viral, inflammatory, or immune-mediated process remains plausible. 

Although autoimmune screening (including ANA and ENA panels) was not performed, such testing could have provided further insight into potential immune-mediated mechanisms. Future cases should consider a comprehensive autoimmune work-up to better delineate immunologic contributions to transient hypothalamic-pituitary dysfunction.

In cases of transient CAI without identifiable causes, the underlying mechanism often remains unclear, presenting a diagnostic challenge. A notable example is seen in polyneuropathy, organomegaly, endocrinopathy, monoclonal plasma cell disorder, and skin changes (POEMS) syndrome, a rare paraneoplastic disorder, where patients can experience CAI despite no triggers [19]. In such cases, temporary dysfunction in the HPA axis leads to a reduction or absence of ACTH secretion, impairing cortisol production. The adrenal glands typically maintain their structural integrity, and function often recovers once the transient dysfunction resolves [2].

The recovery timeline in transient CAI varies. Some patients recover within weeks, while others take up to two years. Reversibility has also been reported following traumatic brain injury, pituitary apoplexy, or steroid withdrawal [1,4,20]. Hence, periodic HPA axis reassessment is crucial in managing these patients.

This case reinforces several clinical considerations. First, clinicians should maintain a high index of suspicion for adrenal insufficiency in patients with nonspecific symptoms, particularly when laboratory findings indicate low cortisol and suppressed ACTH. Second, prompt initiation of glucocorticoid replacement remains essential to alleviate symptoms and prevent adrenal crisis. Equally important is periodic reassessment of adrenal function to confirm recovery and avoid unnecessary prolonged steroid therapy [16,17]. Finally, transient or functional causes of CAI should be considered in the absence of structural pituitary lesions, as spontaneous resolution is possible in select cases.

This case emphasizes the need for clinicians to consider transient idiopathic causes of CAI in patients without clear triggers. It underscores the importance of a comprehensive diagnostic approach, keeping reversible and functional etiologies in mind when HPA axis dysfunction is suspected.

## Conclusions

In summary, this case contributes to the growing but limited literature on transient, idiopathic CAI and underscores the importance of a cautious yet thorough diagnostic approach. Although the patient achieved full recovery with short-term hydrocortisone therapy, the absence of dynamic pituitary testing, autoimmune marker evaluation, and a defined precipitating cause limits definitive identification of the underlying mechanism. Nevertheless, the complete recovery supports the potential reversibility of HPA axis dysfunction. Further studies are warranted to elucidate the pathophysiological basis, mechanisms, and long-term outcomes of idiopathic or reversible CAI.
